# Evolution of endometrial cancer incidence patterns in Hong Kong: A three-decade analysis with future projections

**DOI:** 10.1016/j.heliyon.2024.e40285

**Published:** 2024-11-08

**Authors:** Xinyue Ma, Xiaoming Wu, Jianqiang Du, Haifeng Sun

**Affiliations:** a Department of Medical Oncology, The First Affiliated Hospital of Xi'an Jiaotong University, Xi'an, Shaanxi, China; b The Key Laboratory of Biomedical Information Engineering of Ministry of Education, School of Life Science and Technology, Xi'an Jiaotong University, Xi'an, Shaanxi, China; c Third Department of Medical Oncology, Shaanxi Provincial Cancer Hospital Affiliated to Medical College of Xi'an Jiaotong University, Xi'an, Shaanxi, China

**Keywords:** Endometrial cancer, Incidence, Period analysis, Cohort effect, Demography

## Abstract

**Objective:**

This study provides a comprehensive analysis of endometrial cancer incidence trends in Hong Kong over the past three decades. It aims to evaluate the impact of demographic shifts and epidemiological factors, including age, birth cohort, and diagnosis period, on the incidence rates. The study also projects future trends in endometrial cancer cases up to 2030 and assesses the contributions of these factors using a detailed decomposition approach.

**Material and methods:**

The analysis is based on endometrial cancer data obtained from the Hong Kong Cancer Registry. Age-period-cohort (APC) modeling was utilized to investigate the effects of different age groups, historical periods, and birth cohorts on the changing incidence patterns. The study projects future trends using a Bayesian APC framework, integrating historical data and expert insights for robust predictions. Additionally, a decomposition analysis was conducted to disentangle the contributions of demographic changes (aging and population growth) and epidemiological shifts (risk factors such as obesity and reproductive behaviors) to the increasing cases.

**Results:**

Between 1992 and 2021, there were 19,214 recorded cases of endometrial cancer in Hong Kong. Age-standardized and crude incidence rates showed consistent increases, rising from 7.4 per 100,000 person-years in 1992 to 31.0 per 100,000 in 2020. Incidence trends rose significantly across all age groups, with the highest increase seen in women aged 50–65. Projections indicate that the upward trend will continue, with an estimated 1718 cases by 2030. Demographic factors, particularly population aging, and evolving epidemiological trends contribute jointly to the incidence rise.

**Conclusions:**

The findings reveal a steady increase in endometrial cancer incidence among Hong Kong women, primarily driven by demographic aging and shifts in risk factors. The study underscores the need for targeted public health measures and resource allocation for early detection and effective management strategies, emphasizing the importance of addressing modifiable risk factors such as obesity and reproductive health behaviors.

## 1 Introduction

Endometrial cancer, also known as cancers of the corpus uteri, primarily comprises adenocarcinomas originating from the endometrium [1]. Globally, endometrial cancer ranks as the sixth most frequently diagnosed malignancy and the 14th leading cause of cancer-related mortality among women, with an estimated 420,000 new cases and 98,000 fatalities reported in 2022 [2]. Notably, the incidence rates of endometrial cancer exhibit significant regional variations, with North America, Europe, and Oceania bearing the highest burdens, while Africa and South Central Asia demonstrate lower rates [1,3]. In recent decades, many countries have observed an increasing or stable trend in endometrial cancer incidence [3,4]. Asian countries have also experienced rising incidence, albeit slower than Western countries [5]. Hong Kong, an urbanized East Asian metropolis, is no exception to this trend, as endometrial cancer ranks as the fourth most prevalent cancer among women and has consistently shown an upward trend in incidence over the past three decades, according to data from the Hong Kong Cancer Registry [6].

Endometrial cancer is traditionally categorized into type I and type II [7]. Type I, accounting for over 80 % of cases, is estrogen-dependent, more prevalent, and typically associated with a better prognosis. In contrast, type II, representing 10 %–20 % of cases, is estrogen-independent, less common, and often presents at more advanced stages with a poorer prognosis. Despite their differences, both types share several common etiological factors, including obesity, parity, and oral contraceptive use [7]. The increasing incidence of endometrial cancer can be attributed to the rising prevalence of these established risk factors in the population [8]. Global obesity rates have doubled in less than 30 years [9], emerging as a significant contributor to the increasing incidence of endometrial cancer. A high body mass index (BMI) alone is estimated to contribute to over one-third of worldwide disease cases [10,11]. Additionally, East Asia has witnessed substantial population growth and aging over the past three decades [12], resulting in a larger number and proportion of women entering the age group at a higher risk for endometrial cancer.

Given the evolving exposure to risk and demographic factors, it becomes imperative to scrutinize the variations in endometrial cancer risk across different year periods and birth cohorts. Age-period-cohort (APC) modeling provides a practical approach for discerning the patterns of endometrial cancer incidence [13]. By disentangling the effects of age, year period, and birth cohort, APC modeling allows us to gain valuable insights into the factors that influence the observed trends. Through APC modeling, we can better understand how age, period, and cohort contribute to endometrial cancer risk and identify specific risk factors that play a role in its incidence.

This study aims to provide an in-depth analysis of the evolving patterns and trends in endometrial cancer incidence in Hong Kong over the past three decades. By employing an age-period-cohort (APC) modeling approach, we explore how different age groups, historical periods, and generational cohorts have influenced incidence rates. Additionally, we project future trends in endometrial cancer cases up to the year 2030, integrating demographic and epidemiological factors such as population aging and shifts in risk behaviors. Understanding these dynamics allows us to identify key drivers behind the increase in cases and offers insights for public health policies to implement targeted prevention strategies and early detection measures, ultimately aiming to mitigate the growing burden of endometrial cancer in Hong Kong and other similar urban populations.

## 2 Material and methods

This study analyzed cases of endometrial cancer diagnosed in Hong Kong from 1992 to 2021, utilizing incidence data obtained from the Hong Kong Cancer Registry (HKCaR) [6]. The HKCaR, a well-established population-based registry operational since 1963, is endorsed by the International Agency for Research on Cancer (IARC) for its comprehensive and high-quality data. The registry compiles detailed information on cancer diagnoses reported by hospitals, private clinics, and other healthcare providers across Hong Kong, including year of diagnosis, age group, and cancer classification. To maintain data integrity, HKCaR undergoes regular audits, ensuring consistency and reliability for epidemiological research. In this study, cases of endometrial cancer were identified using the International Classification of Diseases codes (ICD-9 [14] code 182 and ICD-10 [15] code C54). To focus on the most relevant population groups, individuals below the age of 20 were excluded due to the very low incidence rates observed in this demographic.

Demographic data for the study, including population estimates and future projections, were sourced from the United Nations World Population Prospects 2022 Revision [16]. These projections, developed by the Population Division of the Department of Economic and Social Affairs, provided authoritative and standardized demographic inputs essential for evaluating trends in endometrial cancer incidence in the Hong Kong population.

### 2.1 Statistical analyses

To facilitate meaningful comparisons of incidence rates across populations with different age distributions, age-standardized incidence rates were calculated using the direct method, applying the World Health Organization (WHO) standard population from the year 2000 [17]. The study categorized cancer cases and population data into fourteen distinct 5-year age groups (ranging from 20 to 24 years to 85+ years) and six 5-year periods (1992–1996 to 2017–2021) to ensure consistency across both age and time-period analyses. Additionally, nineteen birth cohorts were defined, spanning from 1905 to 1909 to 1995–1999, based on these age and time period groupings.

To analyze the incidence trends, age-period-cohort (APC) models were employed to estimate net drift and local drift. Net drift indicates the annual percentage change in age-adjusted incidence rates over time, while local drift shows the annual percentage change for specific age groups. The study used the earliest period (1992–1996) and the youngest age group (20–24 years) as reference points, presenting period and cohort effects as rate ratios relative to these references. Period effects provide insights into influences affecting all age groups simultaneously, whereas cohort effects reveal generational changes in risk exposure and disease patterns. The APC models were fitted using the National Cancer Institute's Age-Period-Cohort analysis web tool (Biostatistics Branch, National Cancer Institute, Bethesda, MD, USA) [18] to evaluate variable significance, with statistical significance determined via Wald tests at a P-value threshold of <0.05.

For projecting future incidence trends from 2022 to 2030, a Bayesian Age-Period-Cohort (APC) model was applied [19]. This approach integrates historical data, expert inputs, and prior knowledge to estimate uncertainties in the projections. The integrated nested Laplace approximation (INLA) method was used for computational efficiency in fitting the Bayesian models. The R BAPC package (version 0.0.36) [19] was utilized for model fitting and parameter estimation, providing reliable uncertainty estimates for the future projections and generating posterior distributions of model parameters [20].

A meticulous decomposition analysis was conducted to discern the factors influencing changes in incident cases of endometrial cancer in Hong Kong from 1992 to 2030. This analysis aimed to quantify the contributions of population growth, population aging, and age-specific incidence rates, which reflect underlying epidemiological shifts. These epidemiological changes encompass variations in risk factors, such as increasing obesity rates and evolving reproductive behaviors, as well as advancements in healthcare, which are independent of population size or demographic aging. The analysis employed a robust algorithm that is unaffected by the choice of reference year or the order of decomposition, ensuring reliable results across different scenarios [21,22]. Details on the decomposition and projection methodologies are provided in Appendices S1 and S2. We used R [23] with tidyverse (version 2.0.0) to perform data analysis and visualization.

## 3 Results

### 3.1 Trends in endometrial cancer incidence in Hong Kong

**Fig. 1:**
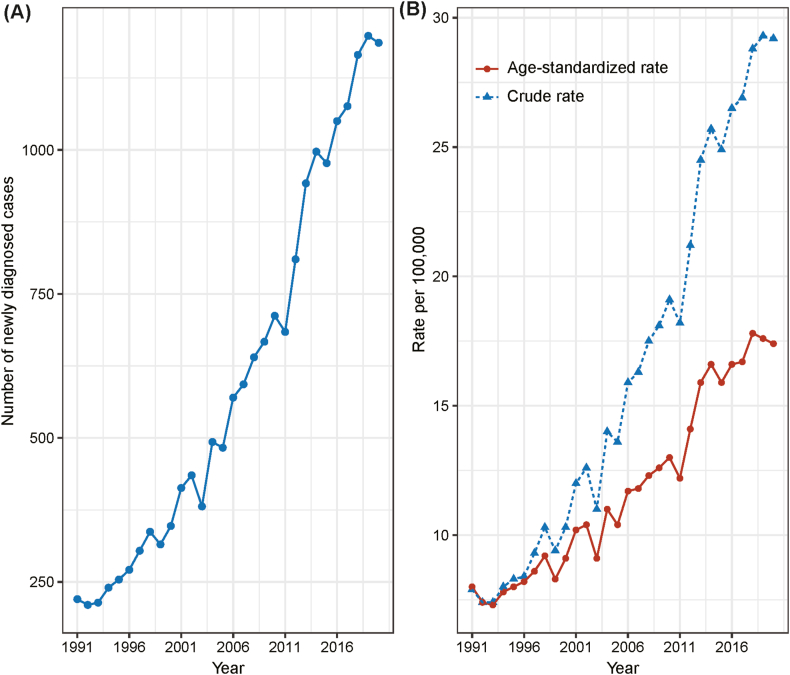
Trends in endometrial cancer incidence and case counts in Hong Kong from 1992 to 2021. (A) Number of newly diagnosed cases. (B) Crude and age-standardized incidence rates over the same period.

From 1992 to 2021, there were 19,214 diagnosed cases of endometrial cancer in Hong Kong, marking a significant increase from 210 cases in 1992 to 1250 cases in 2021 (Fig. 1A). The crude and age-standardized incidence rates exhibited a clear upward trend, rising from 7.4 per 100,000 person-years in 1992 to 31.0 per 100,000 for crude rates and 18.0 per 100,000 for age-standardized rates in 2021 (Fig. 1B). This upward trajectory underscores a notable increase in the disease burden over the study period.

### 3.2 APC modeling

**Fig. 2:**
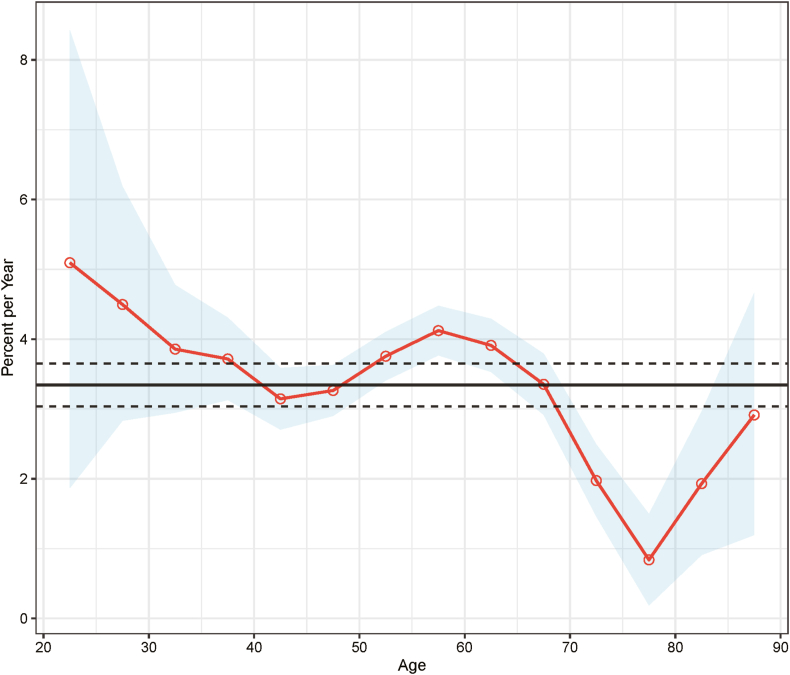
Local and net drift values for endometrial cancer incidence in Hong Kong (1992–2020). The solid black horizontal line represents the net drift, with dashed lines indicating the 95 % confidence intervals. The red curve illustrates the local drift values across age groups, and the shaded area denotes the 95 % confidence intervals for the local drift. (For interpretation of the references to colour in this figure legend, the reader is referred to the Web version of this article.)

The age-period-cohort (APC) analysis revealed a net drift, indicating an annual percentage increase of 3.38 % (95 % confidence interval [CI]: 3.10–3.67) in age-adjusted incidence rates throughout the study period (Fig. 2). Local drift, which measures age-specific changes in incidence trends, showed positive values across all age groups, suggesting a consistent rise in incidence rates. However, variability in the local drift was more pronounced among younger age groups, as indicated by the wider 95 % confidence intervals, reflecting higher uncertainty in these cohorts. The most significant local drift increase occurred in the 50–65 age group, while those older than 65 exhibited comparatively smaller increments (Fig. 2).

**Fig. 3:**
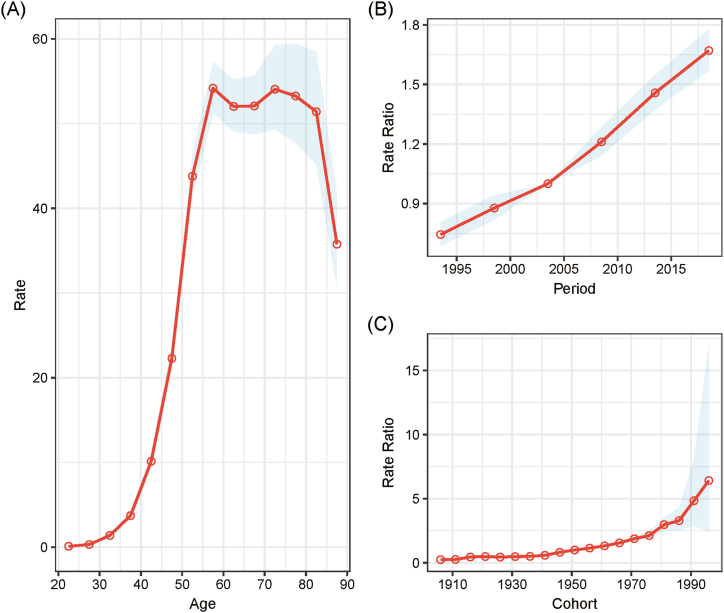
Age, period, and cohort effects on endometrial cancer incidence rates in Hong Kong, including 95 % confidence intervals. (A) Longitudinal curves of fitted age-specific rates for the reference cohort, adjusted for period effects. (B) Rate ratios for each period, adjusted for age and nonlinear cohort effects, relative to the reference period. (C) Rate ratios for each cohort, adjusted for age and nonlinear period effects, relative to the reference cohort.

After adjusting for period deviations, the incidence of endometrial cancer in Hong Kong women followed an age-dependent pattern, peaking around the age of 55, then plateauing before sharply declining after the age of 85 (Fig. 3). The relationship between incidence rates and age was effectively modeled using a second-order polynomial regression, which had an R-squared value of 0.988, indicating 98.8 % of the variance in incidence rates could be explained by the model.

Additionally, the period effect indicated a consistent increase in endometrial cancer risk over time for Hong Kong women, while the cohort effect showed a persistent rise in risk across various generational cohorts (Fig. 3). All parameters tested were found to be statistically significant (Table S1), reinforcing the robustness of the APC model outcomes.

### 3.3 Projection

**Fig. 4:**
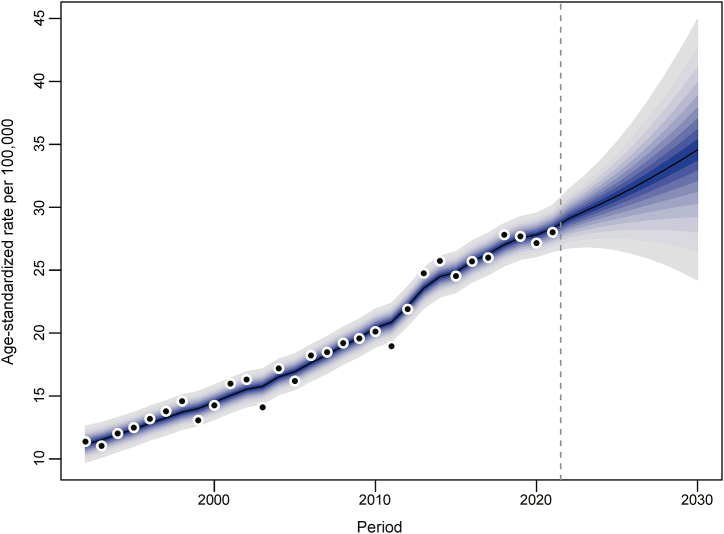
Observed and projected incidence rates of endometrial cancer among women in Hong Kong. The dashed line separates observed data (left) from projected data (right). Each lighter shade of blue indicates a 10 % increment in the confidence interval (CI). (For interpretation of the references to colour in this figure legend, the reader is referred to the Web version of this article.)

Using the Bayesian Age-Period-Cohort (APC) model, the study projected trends in endometrial cancer incidence in Hong Kong from 2022 to 2030. The age-standardized incidence rate is expected to continue its upward trajectory, reflecting a persistent increase in cases among Hong Kong women (Fig. 4). The number of new cases is projected to rise from 1250 in 2021 to 1718 by 2030 (Table S2), with the most significant increase anticipated among women aged 40 to 75.

### 3.4 Decomposition

**Fig. 5:**
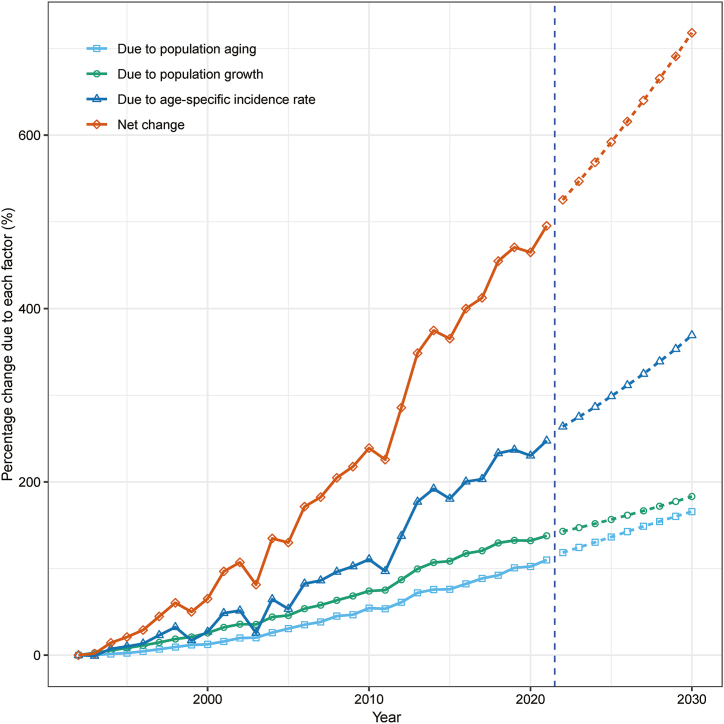
Contribution of population ageing, population growth, and age-specific incidence rate to the changes in endometrial cancer cases in Hong Kong from 1993 to 2030, using 1992 as the reference year. The section to the right of the blue dashed line represents data derived from the decomposition of projected estimates. (For interpretation of the references to colour in this figure legend, the reader is referred to the Web version of this article.)

A decomposition analysis was performed to understand the factors contributing to the surge in endometrial cancer cases. Between 1992 and 2021, there was a significant 495.2 % increase in cases. This rise is attributable to several factors: population aging accounted for 109.9 %, population growth contributed 137.8 %, and epidemiological changes, including shifts in risk factors such as obesity and reproductive behaviors, made up 247.5 % of the increase (Fig. 5, Table S3).

Projections for 2030, based on the decomposition analysis, indicate that demographic and epidemiological factors will continue to drive the increase in endometrial cancer cases among Hong Kong women. Compared to 1992, the number of cases is projected to grow by 1508 cases (a 718.1 % increase) by 2030. This projected increase is broken down as follows: 348 cases (165.7 %) due to population aging, 385 cases (183.2 %) from population growth, and 775 cases (369.2 %) resulting from epidemiological changes (Fig. 5, Table S3). These findings emphasize the substantial impact of evolving risk factors, such as increasing obesity rates and changing reproductive patterns, on the epidemiological component of the incidence rise.

## 4 Discussion

Our study provides critical insights into the rising incidence of endometrial cancer among women in Hong Kong over the past three decades, highlighting an increased risk across both pre- and post-menopausal groups. The analysis attributes this trend to demographic shifts, such as population aging and growth, alongside epidemiological changes, including rising obesity rates and evolving reproductive behaviors. Projections suggest that without intervention, this trend is likely to continue, indicating a sustained rise in cases. These findings emphasize the need for strengthened healthcare infrastructure and comprehensive prevention strategies. Targeted public health initiatives, focusing on modifiable risk factors and promoting early detection, are crucial to mitigate this upward trajectory and reduce the future burden of endometrial cancer in Hong Kong.

Estrogen-related factors are significant contributors to the rising incidence of endometrial cancer in Hong Kong, particularly in the case of Type I tumors, which account for the majority of endometrial cancer cases and are estrogen-dependent [7]. Key factors include both exogenous estrogen exposure, such as hormone replacement therapy (HRT) in peri- and post-menopausal women, and endogenous estrogen exposure, which is influenced by factors like nulliparity, lower parity, early age at menarche, and obesity [4,7,24]. However, it is important to note that the influence of exogenous estrogen exposure, such as HRT, may be relatively limited in Hong Kong compared to Western countries, given the low prevalence of hormone therapy use among Hong Kong women, which stands at less than 3 % [25]. Additionally, lower rates of oral contraceptive (OC) use in Hong Kong and other East Asian countries [[26], [27], [28]], which have been shown to reduce the risk of endometrial cancer, suggest that the impact of OC use on lowering incidence rates in Hong Kong is likely minimal.

Changes in reproductive factors are likely significant contributors to the increasing incidence of endometrial cancer in Hong Kong. The declining fertility rate and a rising prevalence of nulliparity have increased the risk of endometrial cancer among women. Socioeconomic transformations have resulted in a decline in the fertility rate from 45.1 live births per 1000 women in 1991 to 26.5 in 2020 [29]. Additionally, the proportion of women remaining childless has increased, with estimates suggesting that up to 35 % of women born in the mid-to late-1960s will remain without children [30]. These shifting reproductive patterns are likely driving the rapid rise in endometrial cancer incidence observed in Hong Kong.

Given that changes in fertility patterns, including declining birth rates and increasing nulliparity, are significant contributors to the cohort and age effects on endometrial cancer incidence, public health officials and policymakers must address these trends. Health education campaigns should encourage lifestyle changes, such as maintaining a healthy weight, to reduce the risks associated with delayed childbirth and nulliparity. Moreover, implementing early and regular gynecological screenings for women who delay childbearing or remain childless could facilitate early detection of endometrial cancer. Policymakers might also explore fertility-related policies that balance societal and individual preferences for delayed childbirth with the associated health risks. Effectively managing the future burden of endometrial cancer in Hong Kong will require addressing both demographic and reproductive shifts.

Another significant factor contributing to the rising incidence of endometrial cancer is the increasing prevalence of obesity, a well-established risk factor for the disease. Over the past three decades, obesity rates have steadily risen both globally and in Hong Kong [9,31]. According to recent data from the Hong Kong Department of Health's Population Health Survey 2020–22, 26.4 % of Hong Kong women aged 15–84 are classified as obese, with an additional 19.7 % categorized as overweight. The highest prevalence of obesity and overweight was observed in women aged 65–84, with 57.0 % falling into these categories [32]. The increasing obesity rates, combined with changing reproductive patterns, are likely major contributors to the rapid rise in endometrial cancer incidence observed in Hong Kong.

The advancements in diagnostic methods and increased screening practices are potential factors that may have contributed to the observed increase in endometrial cancer incidence. Improved medical technology, including the increased use of ultrasound and endometrial biopsy, may have facilitated earlier detection of endometrial cancer in more recent birth cohorts than earlier cohorts. The influence of these advancements may explain some of the period and cohort effects observed in endometrial cancer trends.

Demographic factors, especially population aging, significantly impact the rising incidence of endometrial cancer in Hong Kong. Over the past few decades, the median age of women in Hong Kong has increased from 31.6 in 1991 to 46.3 in 2020, alongside population growth from 2.81 million to 4.06 million women. These demographic shifts have resulted in a larger proportion of women entering age groups at higher risk for endometrial cancer [33].

The projections and decomposition analysis underscore the significant contributions of demographic and epidemiological factors to the rise in endometrial cancer cases in Hong Kong. Population aging and growth were responsible for a large portion of the increase, but the changes in age-specific incidence rates, driven by shifts in risk factors, played an even more substantial role. The decomposition analysis also revealed important interactions between these factors, amplifying the overall trend. This novel approach to understanding the net change in incidence highlights the complex interplay between demographic shifts and epidemiological factors, offering valuable insights for public health interventions. This demographic trend is likely to be observed in other East Asian countries and regions grappling with population aging and experiencing changes in fertility patterns [34].

These findings highlight the critical need to address the impact of an aging population on the incidence of endometrial cancer. Healthcare systems and policymakers must prioritize the specific healthcare requirements and challenges posed by the increasing number of women entering high-risk age groups. This may include implementing targeted prevention and screening programs, enhancing diagnostic capabilities, and ensuring sufficient healthcare resources are allocated to effectively manage the growing burden of endometrial cancer. Furthermore, similar demographic trends in other East Asian countries and regions call for regional collaboration and the development of comprehensive strategies to manage the rising incidence of endometrial cancer, considering both population aging and changing fertility patterns.

Despite the strengths of our study, several limitations should be acknowledged. First, the Hong Kong Cancer Registry (HKCaR) does not report subtypes of endometrial cancer, limiting our ability to assess the specific impact of each subtype on the overall analysis. However, given that the majority of endometrial cancers are Type I, our findings largely remain relevant. Second, the absence of patient-level data on risk factor exposure prevents a direct analysis of how these factors influence endometrial cancer incidence. Future research incorporating individual-level data would provide a more detailed understanding of the disease's etiology in Hong Kong. Additionally, our projections for future endometrial cancer incidence are based on population estimates from the United Nations' World Population Prospects, which, while valuable, may underestimate the extent of population aging, potentially introducing bias into our projections. Nonetheless, our study offers significant insights into the age-period-cohort associations with endometrial cancer incidence in Hong Kong. By utilizing a comprehensive three-decade dataset and integrating age, period, and cohort effects, we provide robust estimates of the key factors driving the increasing incidence of endometrial cancer among women in Hong Kong.

## 5 Conclusion

Our study highlights the growing risk of endometrial cancer among women in Hong Kong, emphasizing the critical need for improved healthcare infrastructure and effective prevention strategies. The rising incidence rates are primarily attributed to demographic and epidemiological changes, including evolving reproductive patterns, increasing obesity prevalence, and population aging. These findings are not confined to Hong Kong; they provide valuable insights that can inform research and healthcare policies in other populations experiencing similar demographic and epidemiological transitions.

## Key message

Endometrial cancer incidence has been rising in Hong Kong women over time and across generations, and this trend is projected to continue due to demographic and epidemiological changes.

## CRediT authorship contribution statement

**Xinyue Ma:** Writing – original draft, Methodology, Investigation, Data curation, Conceptualization. **Xiaoming Wu:** Validation, Investigation, Data curation, Conceptualization. **Jianqiang Du:** Validation, Methodology, Formal analysis. **Haifeng Sun:** Writing – review & editing, Supervision, Project administration, Investigation, Data curation, Conceptualization.

## Ethics declarations

Ethics approval from the institutional review board and informed consent were not required for this study, as it utilized publicly available, aggregated, and de-identified data, ensuring that no individual patient information was accessed or disclosed.

## Data availability statement

All data used in this study are publicly accessible and can be obtained from the Hong Kong Cancer Registry's website (http://www3.ha.org.hk/cancereg/allages.asp).

## Declaration of AI and AI-assisted technologies in the writing process

During the preparation of this work the authors used the artificial intelligence assistant Kimi, developed by Moonshot AI, in order to improve the readability. After using this service, the authors reviewed and edited the content as needed and take full responsibility for the content of the publication.

## Funding

This work was funded by 10.13039/100018904Beijing Xisike Clinical Oncology Research Foundation under Grant No. Y-QL202102-0175 and Beijing Science and Technology Innovation Medical Development Foundation under Grant No. KC2023-JX-0186-FQ032.

## Declaration of competing interest

The authors declare the following financial interests/personal relationships which may be considered as potential competing interests: Haifeng Sun reports financial support was provided by 10.13039/100018904Beijing Xisike Clinical Oncology Research Foundation. Haifeng Sun reports financial support was provided by Beijing Science and Technology Innovation Medical Development Foundation. If there are other authors, they declare that they have no known competing financial interests or personal relationships that could have appeared to influence the work reported in this paper.
