# Supplementary material for: Evolution of endometrial cancer incidence patterns in Hong Kong: A three-decade analysis with future projections
Source: Heliyon. 2024 Nov 8;10(22):e40285. doi: 10.1016/j.heliyon.2024.e40285 (PMC11693884; doi:10.1016/j.heliyon.2024.e40285)
Supplement: Multimedia component 1 [file mmc1.docx]

**SUPPLEMENTARY MATERIAL**

**Table of content**

**Appendix S1:** Methodology for the decomposition analysis.

**Appendix S2:** Projections of future endometrial cancer incidence using the Bayesian Age-Period-Cohort (APC) model with integrated nested Laplace approximations.

**Table S1:** Results of Wald Chi-square tests for the estimable parameters within the APC model.

**Table S2:** Age-specific estimates of endometrial cancer cases in Hong Kong spanning from 1992 to 2030.

**Table S3:** Breakdown of the impact of population aging, population growth, and changes in age-specific incidence rates on the net increase of endometrial cancer cases in Hong Kong, from 1993 to 2030, using 1992 as a baseline.

**Appendix S1. Methodology for the decomposition analysis.**

The population decomposition algorithm has been described in detail in the papers by Cheng et al. [1,2].

Briefly, take the difference in newly diagnosed endometrial cancer cases between 1992 and 2021 in Hong Kong. We can decompose the net change of incident cases into the contribution of population growth, population aging, and age-specific incidence rate.

The age groups were divided using 5-year increments from 20-24 years to 85 plus (we included older people aged ≥85 years as the 85-89 years age group, which was recorded as only one group in the database). Let *d_ij_*, *n_ij_*, *m_ij_* and *s_ij_* denote the incident cases, population size, age-specific rate of incidence, and population proportion in the *i*^th^ age group of the year *j*, respectively, (*i* = 1, 2, …,14; *j* = 1, 2). Let *D*_1_ and *D*_2_, *N*_1_ and *N*_2_, *P*_1_ and *P*_2_ represent the total incident cases, population size, and crude rate of incidence in 1992 and 2021.

Using *M_p_*, *M_a,_* and *M_m_* to represent the main effects of the changes in population size, age structure, and incidence rate, and *I_pa_*, *I_pm_*, *I_am,_* and *I_pam_* to represent their two-way and three-way interactions, respectively. In the case of 1992 as the reference year, these terms are calculated as follows:

$M_{p}=\sum_{i=1}^{12} {{\left( N_{2}-N_{1} \right)s}_{i1}m}_{i1}$

$M_{a}=\sum_{i=1}^{12} N_{1}\left( s_{i2}-s_{i1} \right)m_{i1}$

$M_{m}=\sum_{i=1}^{12} {N_{1}s}_{i1}\left( m_{i2}-m_{i1} \right)$

$I_{pa}=\sum_{i=1}^{12} \left( N_{2}-N_{1} \right)\left( s_{i2}-s_{i1} \right)m_{i1}$

$I_{pm}=\sum_{i=1}^{12} \left( N_{2}-N_{1} \right)s_{i1}\left( m_{i2}-m_{i1} \right)$

$I_{am}=\sum_{i=1}^{12} N_{1}\left( s_{i2}-s_{i1} \right)\left( m_{i2}-m_{i1} \right)$

$I_{pam}=\sum_{i=1}^{12} \left( N_{2}-N_{1} \right)\left( s_{i2}-s_{i1} \right)\left( m_{i2}-m_{i1} \right)$

Here, a simplification needs to be made, assuming that the interactions are equally distributed, then the contribution of the three factors can be calculated as follows:

$A{=M}_{a}+½I_{am}+½I_{pa}+⅓I_{pam}$

$P{=M}_{p}+½I_{pm}+½I_{pa}+⅓I_{pam}$

$M{=M}_{m}+½I_{pm}+½I_{am}+⅓I_{pam}$

Here, *A* represents the contribution of population aging, *P* represents the contribution of population growth, *M* represents the contribution of the age-specific death rate, and net change represents total change. The contribution of each factor divided by *D_1_* and multiplied by 100 is the percentage of the respective contribution.

**References**

1. Cheng X, Yang Y, Schwebel DC, Liu Z, Li L, Cheng P et al. Population ageing and mortality during 1990-2017: A global decomposition analysis. PLoS Med 2020;17:e1003138.

2. Cheng X, Tan L, Gao Y, Yang Y, Schwebel DC , Hu G. A new method to attribute differences in total deaths between groups to population size, age structure and age-specific mortality rate. PLoS One 2019;14:e0216613.

**Appendix S2. Projections of future endometrial cancer incidence using the Bayesian Age-Period-Cohort (APC) model with integrated nested Laplace approximations.**

The future incident cases of endometrial cancer in Hong Kong were projected using the Bayesian age-period-cohort analysis with integrated nested Laplace approximations (INLA). Compared to the classical age-period-cohort model, the Bayesian approach attributes separate effects to age, period, and cohort, and extrapolates these effects to make projections. The Bayesian approach leads to more precise estimates of incidence rates than the corresponding maximum likelihood estimates used in the classical model. In addition, since the Bayesian approach does not depend on strong parametric assumptions like the classical approach, it is the only current method to achieve nonarbitrary and sensible projections.

Based on the expectation that effects adjacent in time might be similar, the Bayesian inference in age-period-cohort model applies the second-order random walk (RW2) for smoothing priors of age, period, and cohort effects and to project posterior incidence rates. According to this model, each point of effect is predicted by linear extrapolation from its two immediate predecessors plus a random variance from a normal distribution with a mean zero. The INLA is used with this Bayesian age-period-cohort model to approximate the marginal posterior distributions avoiding any mixing and convergence issues introduced by Markov chain Monte Carlo (MCMC) sampling techniques traditionally used in the Bayesian approach. The Bayesian age-period-cohort analysis was conducted by an R-package BAPC available from R-forge (http://r-forge.r-project.org/).

We used inverse gamma priors with shape parameter a = 1 and rate parameter b = 0.00005 for the RW2 variance parameters of the time effects (age, period, and cohort) and an inverse gamma prior with a = 1 and b = 0. 005 for the overdispersion variance. It has been found that the results of age-specific and age-standardized predictions have little prior sensitivity.

We prepared age-specific incident cases of endometrial cancer (from 1992 to 2021) and Hong Kong population data (from 1992 to 2030), followed by a 9-year (from 2022 to 2030) retrospective projection using the BAPC function in the R package BAPC.

**References**

1. Riebler, A. and L. Held, Projecting the future burden of cancer: Bayesian age-period-cohort analysis with integrated nested Laplace approximations. Biometrical Journal, 2017. 59(3): p. 531-549.

2. Jacobs, D., et al., Assessment of Age, Period, and Birth Cohort Effects and Trends in Merkel Cell Carcinoma Incidence in the United States. Jama Dermatology, 2021. 157(1): p. 59-65.

**Table S1. Results of Wald Chi-square tests for the estimable parameters within the APC model.**

| Null Hypothesis | Chi-square | P-value |
| --- | --- | --- |
| NetDrift = 0 | 565.2 | 0 |
| All Age Deviations = 0 | 3218.6 | 0 |
| All Period RR = 1 | 639.7 | 0 |
| All Cohort RR = 1 | 1042.9 | 0 |
| All Local Drifts = Net Drift | 108.1 | 0 |

**Table S2. Age-specific estimates of endometrial cancer cases in Hong Kong spanning from 1992 to 2030.**

| Year |  | Number of age-specific endometrial cancer cases | | | | | | | | | | | | | |
| --- | --- | --- | --- | --- | --- | --- | --- | --- | --- | --- | --- | --- | --- | --- | --- |
|  | 20-24 | 25-29 | 30-34 | 35-39 | 40-44 | 45-49 | 50-54 | 55-59 | 60-64 | 65-69 | 70-74 | 75-79 | 80-84 | 85+ | Total |
| 1992 | 0 | 2 | 13 | 11 | 20 | 29 | 30 | 26 | 31 | 21 | 13 | 8 | 5 | 1 | 210 |
| 1993 | 1 | 2 | 7 | 12 | 21 | 15 | 33 | 27 | 38 | 21 | 16 | 14 | 4 | 3 | 214 |
| 1994 | 1 | 1 | 3 | 11 | 27 | 30 | 25 | 41 | 27 | 28 | 18 | 16 | 8 | 4 | 240 |
| 1995 | 0 | 2 | 5 | 17 | 31 | 32 | 36 | 32 | 29 | 21 | 21 | 18 | 6 | 4 | 254 |
| 1996 | 0 | 0 | 3 | 6 | 28 | 49 | 40 | 34 | 39 | 31 | 19 | 16 | 2 | 4 | 271 |
| 1997 | 1 | 3 | 11 | 16 | 24 | 50 | 48 | 30 | 31 | 30 | 31 | 11 | 12 | 6 | 304 |
| 1998 | 0 | 1 | 5 | 24 | 31 | 58 | 51 | 34 | 34 | 31 | 24 | 22 | 16 | 6 | 337 |
| 1999 | 0 | 1 | 3 | 17 | 34 | 59 | 47 | 31 | 30 | 24 | 32 | 28 | 8 | 1 | 315 |
| 2000 | 1 | 1 | 7 | 16 | 29 | 57 | 70 | 43 | 29 | 32 | 26 | 19 | 11 | 6 | 347 |
| 2001 | 1 | 3 | 9 | 18 | 48 | 61 | 80 | 47 | 33 | 39 | 34 | 19 | 10 | 11 | 413 |
| 2002 | 0 | 2 | 16 | 22 | 50 | 76 | 93 | 51 | 39 | 24 | 25 | 19 | 12 | 6 | 435 |
| 2003 | 0 | 0 | 6 | 18 | 36 | 73 | 78 | 61 | 37 | 27 | 24 | 7 | 9 | 5 | 381 |
| 2004 | 1 | 0 | 4 | 21 | 45 | 67 | 104 | 86 | 37 | 43 | 27 | 24 | 25 | 9 | 493 |
| 2005 | 2 | 2 | 9 | 23 | 50 | 92 | 109 | 64 | 38 | 32 | 29 | 18 | 11 | 4 | 483 |
| 2006 | 1 | 5 | 8 | 29 | 56 | 84 | 110 | 100 | 36 | 40 | 38 | 35 | 19 | 9 | 570 |
| 2007 | 0 | 7 | 6 | 19 | 55 | 87 | 124 | 104 | 55 | 37 | 37 | 25 | 18 | 19 | 593 |
| 2008 | 0 | 5 | 10 | 21 | 66 | 98 | 146 | 110 | 58 | 27 | 40 | 30 | 19 | 10 | 640 |
| 2009 | 0 | 3 | 7 | 23 | 60 | 82 | 157 | 122 | 73 | 40 | 34 | 32 | 22 | 12 | 667 |
| 2010 | 1 | 1 | 14 | 20 | 60 | 122 | 165 | 129 | 78 | 33 | 27 | 28 | 19 | 15 | 712 |
| 2011 | 1 | 0 | 6 | 31 | 59 | 112 | 150 | 128 | 82 | 38 | 22 | 27 | 15 | 13 | 684 |
| 2012 | 5 | 3 | 12 | 29 | 51 | 109 | 186 | 165 | 107 | 47 | 27 | 33 | 19 | 17 | 810 |
| 2013 | 1 | 4 | 16 | 24 | 54 | 129 | 226 | 191 | 132 | 57 | 38 | 34 | 22 | 14 | 942 |
| 2014 | 1 | 0 | 26 | 32 | 82 | 129 | 238 | 213 | 118 | 71 | 29 | 24 | 16 | 18 | 997 |
| 2015 | 0 | 6 | 9 | 30 | 77 | 117 | 209 | 216 | 129 | 80 | 36 | 25 | 31 | 12 | 977 |
| 2016 | 0 | 4 | 14 | 41 | 54 | 129 | 229 | 216 | 147 | 107 | 37 | 30 | 21 | 21 | 1050 |
| 2017 | 1 | 1 | 12 | 33 | 62 | 165 | 227 | 205 | 143 | 106 | 50 | 28 | 22 | 21 | 1076 |
| 2018 | 2 | 5 | 20 | 47 | 87 | 133 | 235 | 245 | 147 | 98 | 64 | 29 | 27 | 26 | 1165 |
| 2019 | 2 | 5 | 13 | 32 | 75 | 134 | 228 | 249 | 170 | 130 | 74 | 29 | 34 | 23 | 1198 |
| 2020 | 2 | 3 | 20 | 34 | 72 | 140 | 204 | 258 | 176 | 127 | 88 | 26 | 19 | 17 | 1186 |
| 2021 | 1 | 2 | 19 | 45 | 75 | 141 | 218 | 238 | 180 | 149 | 98 | 35 | 21 | 28 | 1250 |
| 2022 | 1 | 4 | 15 | 41 | 86 | 148 | 236 | 260 | 197 | 137 | 96 | 42 | 25 | 25 | 1313 |
| 2023 | 1 | 4 | 15 | 41 | 90 | 152 | 244 | 258 | 206 | 146 | 102 | 50 | 24 | 25 | 1358 |
| 2024 | 1 | 4 | 15 | 41 | 94 | 155 | 250 | 256 | 216 | 156 | 107 | 58 | 25 | 26 | 1404 |
| 2025 | 1 | 4 | 15 | 42 | 97 | 161 | 256 | 257 | 223 | 165 | 112 | 67 | 26 | 27 | 1453 |
| 2026 | 1 | 4 | 15 | 42 | 99 | 169 | 260 | 262 | 229 | 173 | 118 | 76 | 28 | 27 | 1503 |
| 2027 | 1 | 4 | 15 | 41 | 101 | 178 | 265 | 270 | 231 | 180 | 125 | 83 | 32 | 28 | 1554 |
| 2028 | 1 | 4 | 16 | 41 | 101 | 188 | 271 | 278 | 230 | 188 | 134 | 88 | 38 | 29 | 1607 |
| 2029 | 1 | 4 | 15 | 41 | 101 | 197 | 279 | 286 | 228 | 198 | 143 | 93 | 45 | 30 | 1661 |
| 2030 | 1 | 4 | 15 | 41 | 102 | 203 | 290 | 293 | 230 | 205 | 152 | 98 | 52 | 32 | 1718 |

**Table S3. Breakdown of the impact of population aging, population growth, and changes in age-specific incidence rates on the net increase of endometrial cancer cases in Hong Kong, from 1993 to 2030, using 1992 as a baseline.**

| year | Population aging(%) | Population growth(%) | Epidemiological change(%) | Net change(%) |
| --- | --- | --- | --- | --- |
| 1993 | 0 (0.1) | 6 (2.7) | -2 (-0.9) | 4 (1.9) |
| 1994 | 3 (1.4) | 12 (5.5) | 15 (7.4) | 30 (14.3) |
| 1995 | 5 (2.4) | 18 (8.4) | 21 (10.1) | 44 (21.0) |
| 1996 | 9 (4.4) | 24 (11.3) | 28 (13.3) | 61 (29.0) |
| 1997 | 15 (7.0) | 31 (14.7) | 48 (23.1) | 94 (44.8) |
| 1998 | 20 (9.4) | 39 (18.7) | 68 (32.4) | 127 (60.5) |
| 1999 | 25 (11.9) | 44 (21.0) | 36 (17.1) | 105 (50.0) |
| 2000 | 26 (12.6) | 54 (25.7) | 57 (27.0) | 137 (65.2) |
| 2001 | 33 (15.9) | 68 (32.2) | 102 (48.6) | 203 (96.7) |
| 2002 | 42 (19.9) | 75 (35.8) | 108 (51.5) | 225 (107.1) |
| 2003 | 42 (20.2) | 75 (35.5) | 54 (25.7) | 171 (81.4) |
| 2004 | 55 (26.1) | 93 (44.1) | 136 (64.6) | 283 (134.8) |
| 2005 | 64 (30.7) | 96 (45.9) | 112 (53.4) | 273 (130.0) |
| 2006 | 74 (35.2) | 113 (53.7) | 173 (82.5) | 360 (171.4) |
| 2007 | 81 (38.4) | 121 (57.8) | 181 (86.2) | 383 (182.4) |
| 2008 | 95 (45.2) | 133 (63.4) | 202 (96.2) | 430 (204.8) |
| 2009 | 98 (46.8) | 144 (68.4) | 215 (102.5) | 457 (217.6) |
| 2010 | 114 (54.4) | 156 (74.1) | 232 (110.6) | 502 (239.0) |
| 2011 | 113 (53.6) | 158 (75.1) | 204 (97.0) | 474 (225.7) |
| 2012 | 128 (61.1) | 183 (87.1) | 289 (137.5) | 600 (285.7) |
| 2013 | 151 (72.0) | 209 (99.6) | 372 (177.0) | 732 (348.6) |
| 2014 | 159 (75.8) | 225 (107.0) | 403 (192.0) | 787 (374.8) |
| 2015 | 160 (76.1) | 228 (108.5) | 379 (180.6) | 767 (365.2) |
| 2016 | 173 (82.3) | 247 (117.4) | 421 (200.3) | 840 (400.0) |
| 2017 | 186 (88.6) | 253 (120.5) | 427 (203.2) | 866 (412.4) |
| 2018 | 194 (92.3) | 272 (129.5) | 489 (233.0) | 955 (454.8) |
| 2019 | 212 (100.9) | 278 (132.5) | 498 (237.1) | 988 (470.5) |
| 2020 | 215 (102.3) | 278 (132.2) | 484 (230.3) | 976 (464.8) |
| 2021 | 231 (109.9) | 289 (137.8) | 520 (247.5) | 1040 (495.2) |
| 2022 | 249 (118.4) | 300 (143.0) | 554 (263.8) | 1103 (525.2) |
| 2023 | 261 (124.4) | 309 (147.2) | 578 (275.1) | 1148 (546.7) |
| 2024 | 274 (130.4) | 319 (151.8) | 601 (286.4) | 1194 (568.6) |
| 2025 | 287 (136.5) | 329 (156.6) | 628 (298.8) | 1243 (591.9) |
| 2026 | 300 (142.6) | 339 (161.6) | 654 (311.5) | 1293 (615.7) |
| 2027 | 312 (148.8) | 350 (166.6) | 682 (324.6) | 1344 (640.0) |
| 2028 | 324 (154.3) | 361 (171.9) | 712 (339.0) | 1397 (665.2) |
| 2029 | 337 (160.2) | 372 (177.4) | 742 (353.3) | 1451 (691.0) |
| 2030 | 348 (165.7) | 385 (183.2) | 775 (369.2) | 1508 (718.1) |
